# The Combination of 2′-Fucosyllactose with Short-Chain Galacto-Oligosaccharides and Long-Chain Fructo-Oligosaccharides that Enhance Influenza Vaccine Responses Is Associated with Mucosal Immune Regulation in Mice

**DOI:** 10.1093/jn/nxz006

**Published:** 2019-04-24

**Authors:** Ling Xiao, Phillip A Engen, Thea Leusink-Muis, Ingrid van Ark, Bernd Stahl, Saskia A Overbeek, Johan Garssen, Ankur Naqib, Stefan J Green, Ali Keshavarzian, Gert Folkerts, Belinda van't Land

**Affiliations:** 1 Utrecht University, Faculty of Science, Department of Pharmaceutical Sciences, Division of Pharmacology, Utrecht, The Netherlands; 2 Department of Internal Medicine, Division of Digestive Diseases and Nutrition, Rush University Medical Center, Chicago, IL USA; 3 Department of Pharmacology, Division of Physiology, Rush University Medical Center, Chicago, IL USA; 4 Danone Nutricia Research, Departments of Immunology/Human Milk Research & Analytical Science, Utrecht, The Netherlands; 5 Sequencing Core, Research Resources Center,University of Illinois at Chicago, Chicago, IL USA; 6 Department of Biological Sciences, University of Illinois at Chicago, Chicago, IL USA; 7 University Medical Center Utrecht, The Wilhelmina Children's Hospital, Laboratory of Translational Immunology, Utrecht, The Netherlands

**Keywords:** oligosaccharides, 2′-fucosyllactose, influenza vaccine, gut microbiota, short-chain fatty acids

## Abstract

**Background:**

A critical role for host-microbe interactions and establishment of vaccine responses has been postulated. Human milk oligosaccharides, of which 2′-fucosyllactose (2′FL) is the most prevalent, are known to alter host-associated microbial communities and play a critical role in the immunologic development of breastfed infants.

**Objectives:**

Dietary supplementation with a combination of 2′FL and prebiotic short-chain (sc) galacto-oligosaccharides (GOS) and long-chain (lc) fructo-oligosaccharides (FOS) was employed to examine human milk oligosaccharide effects on immune responsiveness, within a murine influenza vaccination model.

**Methods:**

Female mice (6 wk old, C57Bl/6JOlaHsd) were fed either control diet (CON) or scGOS/lcFOS/2′FL-containing diet (GF2F) for 45 d. After starting dietary intervention (day 14), mice received a primary influenza vaccination (day 0) followed by a booster vaccination (day 21), after which ear challenges were conducted to measure vaccine-specific delayed type hypersensitivity (DTH). Serum immunoglobulin (Ig) levels, fecal and cecal microbial community structure, short-chain fatty acids, host intestinal gene expression and cellular responses in the mesenteric lymph nodes (MLNs) were also measured.

**Results:**

Relative to CON, mice fed the GF2F diet had increased influenza vaccine–specific DTH responses (79.3%; *P* < 0.01), higher levels of both IgG1 (3.2-fold; *P* < 0.05) and IgG2a (1.2-fold; *P* < 0.05) in serum, and greater percentages of activated B cells (0.3%; *P* < 0.05), regulatory T cells (1.64%; *P* < 0.05), and T-helper 1 cells (2.2%; *P* < 0.05) in their MLNs. GF2F-fed mice had elevated cecal butyric (*P* < 0.05) and propionic (*P* < 0.05) acid levels relative to CON, which correlated to DTH responses (*R*^2 ^= 0.22; *P* = 0.05 and *R*^2 ^= 0.39; *P* < 0.01, respectively). Specific fecal microbial taxa altered in GF2F diet fed mice relative to CON were significantly correlated with the DTH response and IgG2a level increases.

**Conclusions:**

Dietary GF2F improved influenza vaccine–specific T-helper 1 responses and B cell activation in MLNs and enhanced systemic IgG1 and IgG2a concentrations in mice. These immunologic changes are correlated with microbial community structure and metabolites.

## Introduction

In addition to the provision of clean water and hygiene measures, vaccinations are an important public health intervention providing protection against serious infections (1). Vaccination efficiency is assessed via the humoral immune response, which is linked to proper B cell memory development (2). Efficient antigen presentation by dendritic cells (DCs) is also an important effect because this leads to adaptive T cell responses that mediate vaccine-induced protection, ensuring induction of high-affinity antibodies and establishment of immune memory (3). The populations most susceptible to infections (e.g., infants, preterm born infants, immunocompromised individuals, as well as the elderly), however, often show a limited responsiveness to vaccination due to their immature or weakened immune systems (4). Alternative strategies for improving vaccine efficacy in these populations are needed.

The gastrointestinal tract microbiota is one of the environmental factors shaping humoral and cellular immune responses in early life (5). Germ-free and antibiotic-treated mice show a significant impairment of antibody responses to influenza vaccination (6), and this response can be restored by reconstitution of the gut microbiota (7). At the level of the cellular response to antigens, DC responses and influenza-specific CD4+ and CD8+ T cell responses have been reported to be influenced by specific commensal microbiota (8). These data indicate that host-microbe interactions play a critical role in establishing vaccine responses. Several mechanisms through which vaccine responsiveness can be influenced by microbiota have been postulated. For example, flagellin produced by gut microbiota can support vaccine-specific antibody responses through TLR5-mediated signaling (6). In addition, gut microbiota can influence vaccine responses through the production of metabolites such as SCFAs. SCFA production through microbial fermentation of carbohydrates [nondigestible dietary fibers, prebiotic oligosaccharides, or human milk oligosaccharides (HMOS), or a combination of these] occurs predominantly in the colon (9). We recently showed that dietary supplementation with HMOS isolated from human milk increased the level of SCFAs in nonobese diabetic mice (10). SCFAs are known to have immunomodulatory effects on various cell types, including B cells (11), DCs (12), and T cells (13). SCFAs derived from dietary fibers have been shown to regulate gene expression and enhance plasma B cell differentiation and energy metabolism, thereby promoting antibody responses in mice (11). HMOS and prebiotic oligosaccharides can have both direct immune modulatory effects and indirect effects through the regulation of gut microbiota structure and microbial metabolites. Thus, we hypothesize that dietary supplementation with HMOS or prebiotic oligosaccharides, or a combination of both, can enhance vaccine-induced humoral and cellular immune responses. Indeed, dietary supplementation with a specific HMOS 2′-fucosyllactose (2′FL) (14) and prebiotic short-chain galacto-oligosaccharides/long-chain fructo-oligosaccharides (scGOS/lcFOS) (15, 16) has been shown to improve influenza vaccine responses in mice, respectively. The role of 2′FL-induced microbiota modulation in relation to vaccine efficacy remains unknown, and only a limited association exists between microbiota changes induced by scGOS/lcFOS and vaccination response (16). Here, we examined whether dietary intervention with a combination of 2′FL and prebiotic scGOS/lcFOS (GF2F) was capable of influencing vaccine-specific immune responses, and investigated whether there is a causal link between alterations of gut microbiota (structure and metabolism) on immune responses induced by GF2F.

## Methods

### Mice

C57Bl/6JOlaHsd mice (5 wk old, female) were purchased from Envigo and housed in the animal facility at Utrecht University. Mice were kept under standard conditions, with a 12-h/12-h dark/light cycle and ad libitum access to food and water. The animals were fed standard diets and received routine care for 1 wk upon arrival, before the start of the dietary intervention. All experiments were approved by the Animal Ethics Committee of Utrecht University (approval DEC 2015.II.243.038).

### Vaccination and dietary intervention protocol

**FIGURE 1:**
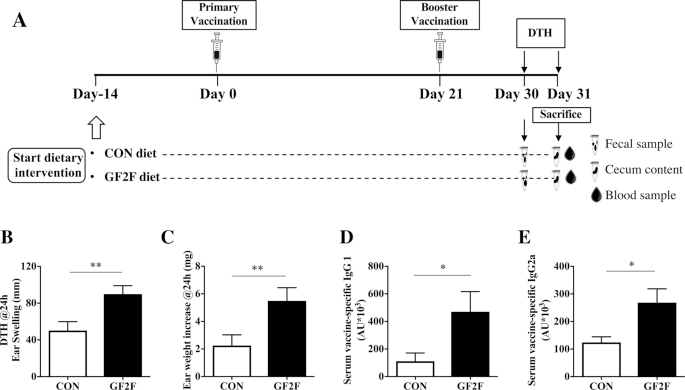
Effects of GF2F diet. (A) Schematic overview of the experimental design. Effects of GF2F diet on influenza vaccine–specific (B) DTH response, (C) ear weight increase, and (D) IgG1 and (E) IgG2a concentrations in serum of vaccinated mice at day 31. DTH response and ear weight were measured 24 h after ear challenge. IgG1 and IgG2a levels in serum were measured by means of ELISA. The Mann-Whitney *U*-test was used. Data are presented as mean ± SEM for *n* = 8–9/group in panels B–E. Significantly different from CON: *P < 0.05; **P < 0.01. AU, arbitrary unit; CON, control; DTH, delayed type hypersensitivity; GF2F, scGOS/lcFOS/2′FL (2′FL, 2′-fucosyllactose; FOS, fructo-oligosaccharides; GOS, galacto-oligosaccharides; lc, long chain; sc, short chain).

The vaccination and dietary intervention protocols employed in this study have been described previously (14). One week after arriving at the facility, 6-wk-old female C57Bl/6JOlaHsd mice were provided with either a control (CON) AIN93G diet (*n* = 9) or the prebiotic diet supplemented with 2.2% GF2F (SNIFF Spezialdiäten GmbH) until the last day of the experiment (**Figure 1**A). GF2F comprises 2′FL (produced by bacterial fermentation and obtained at >90% purity) combined with an equal amount of scGOS (Friesland Campina) and lcFOS (Orafti) a 9:1 (wt/wt) ratio (*n* = 9). A total of 2.2% (wt/wt) of carbohydrates present in the CON diet was exchanged for GF2F. At day 0 (14 d after start of dietary intervention) the mice received a primary vaccination (subcutaneous 125 µL Influvacc; Abbott Biologicals BV), followed by a secondary booster vaccination (subcutaneous 125 µL Influvacc) on day 21. At the end of the experiment, ear swelling [24-h delayed type hypersensitivity (DTH), set at day 30, measured at day 31] and cellular and humoral immune responses (day 31) were measured as previously described (14). Vaccine-specific IgG1 and IgG2a serum levels were determined using ELISA as described previously (14). Fecal (day 30) and cecal (day 31) microbial content and SCFAs were measured as described below. In addition, regarding the effects on the gut's bacterial profile the contribution of individual oligosaccharides in the GF2F mixture were assessed in feces from mice (*n* = 9/group) fed either 2′FL or scGOS at 0.25%, 0.5%, 1%, 2.5%, 5%.

### Flow cytometry of immune cells

**FIGURE 2:**
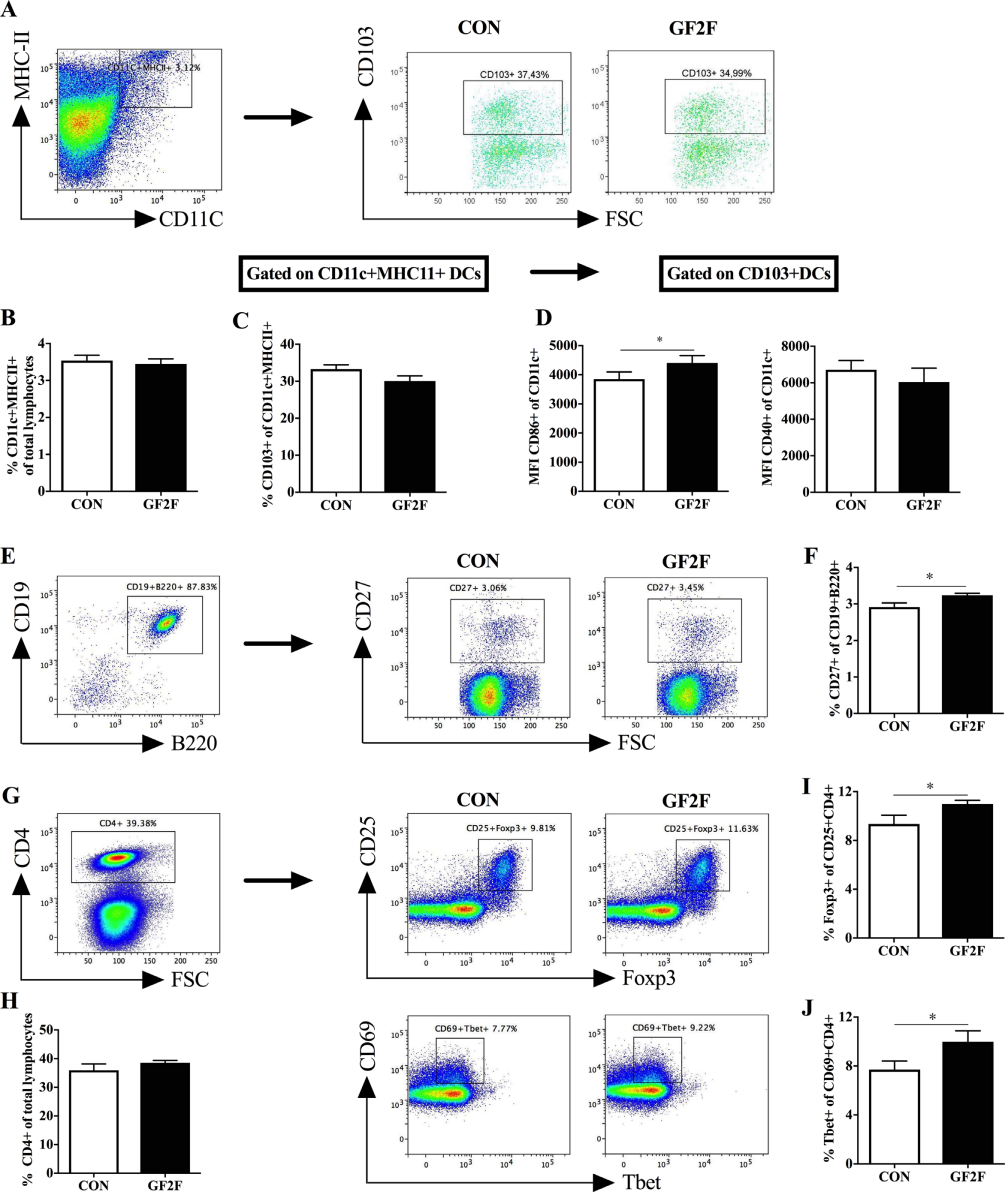
Effects of GF2F diet on the percentage of (B) CD11c+ MHCII+ DCs; (C) CD103+ DCs of CD11c+ MHCII+ cells; (D) maturation status of CD013+ DCs by CD86+ and CD40+ MFI; (F) the percentage of CD27+ B cells of total CD19+ B220+ B cells; (H) percentage of CD4+ cells in the total lymphocytes; (I) percentage of CD25+ Foxp3+ Treg; and (J) CD69+ Tbet+ Th1 of CD4+ T cells in the MLNs of vaccinated mice. Gating and representative plots of (A) CD103+ DCs (CD11c+ MHCII+); (E) CD27+ B cells; (G) Foxp3+ Treg and Tbet+ Th1 cells in the MLNs from CON and GF2F groups are shown. The Mann-Whitney *U* test was used. Data are presented as mean ± SEM for *n* = 8–9/group. Significantly different from CON: *P < 0.05; **P < 0.01. CON, control; DC, dendritic cell; GF2F, scGOS/lcFOS/2′FL (2′FL, 2′-fucosyllactose; FOS, fructo-oligosaccharides; GOS, galacto-oligosaccharides; lc, long chain; sc, short chain); MFI, medium fluorescence intensity; MLN, mesenteric lymph node.

Freshly isolated mesenteric lymph nodes (MLNs) were analyzed by flow cytometry (14). Identification of migratory DCs in the MLNs was performed by gating CD103+ cells out of CD11c+ MHC-II cells in the MLNs (the gating strategy is shown in **Figure 2**A). Cells obtained and resuspended in PBS with 1% bovine serum albumin were incubated with antimouse CD16/CD32 (Mouse BD Fc Block; BD Pharmingen) for 20 min on ice to block nonspecific binding sites. For surface staining, cells were incubated with CD4-PerCp-Cy5.5, CD69-PE, CD25-AlexaFluor488, CD11c-PerCp-Cy5.5, CD103-APC, CD40-FITC, CD86-PE-cy7, MHCII-PE, CD3-Percy5.5, CD27-PE, CD19-APC, B220-FITC (eBiosciences). Foxp3-PE-cy7, and Tbet-APC (eBioscience) were used for intracellular staining. Staining and flow cytometry were performed as described previously (14).

### Generation and treatment of bone marrow–derived dendritic cells

Induction of immature bone marrow–derived dendritic cells (BMDCs) (iDCs) was performed as described previously (10). iDCs (1 × 10^6^) were treated either with medium only (negative CON), medium containing 2 mM acetate (Ace), 2 mM butyrate (But), 2 mM propionate (Pro), or a mixture of SCFAs (all from Sigma-Aldrich) for 5 h. The treated iDCs were then loaded with 0.9 μg/mL vaccine by directly adding Influvacc into the cell culture plate, and cultured for 24 h thereafter. Phenotypes of iDCs were identified by flow cytometry after staining with cell viability dye: CD11c, CD40, CD80, CD86, MHC-II antibodies.

### Ex vivo restimulation of splenocytes with vaccine-loaded BMDCs treated with or without SCFAs

To establish antigen-specific cellular responses, and to investigate the effects of SCFAs on the vaccine-specific antigen-presenting capacity of BMDCs, a separate small animal experiment was performed to obtain spleen cells from nonvaccinated and vaccinated mice. Fresh spleens were removed aseptically from 3 sham mice and 6 vaccinated mice (on CON diet). Single cell suspensions were labeled with cell trace dye CFSE (Thermo Fisher) according to the manufacturer's instructions at a final concentration of 1 µM. Fluorescein isothiocyanate–positive cells were acquired and cocultured with SCFA-treated DCs (iDCs loaded with influenza virus or control iDCs)at ratio of 10:1, in 96-well U-bottomed culture plates for 5 d at 37°C, 5% CO_2_. The mix of cocultured cells was collected after 5 d of incubation, and stained with viability dye, as well as CD4-APC and CD8-PE (eBioscience), to allow quantification of vaccine-specific CD4+ and CD8+ proliferation of splenocytes by flow cytometry.

### qPCR analysis

Quantitative analysis of messenger RNAs was performed as described previously (14). Total RNA was extracted from ileum samples obtained from vaccinated mice and cDNA syntheses were conducted. Custom-designed primers sets targeting the genes *Cd40, Cd80, Cd86, Il1a, Il1b, Il12p40, Cxcl9, Cxcl10, Cldn1, Cldn2, Cldn3, Zo1, Tnfa, Tgfb*, and GAPDH were synthesized (Biolegio; the primer sequences and annealing temperatures are listed in **Supplemental Table 1**). qPCR reactions were conducted in 10 µL reaction volumes (8.8 µL mix + 1.2 µL cDNA), with primers at a final concentration of 300 nM. All reactions were performed in in a 96-well plate (HSP9601; Bio-Rad), and covered with optical film (MSB1001; Bio-Rad). Reactions were conducted in a CFX96 Real-Time System C1000 Thermal Cycler (Bio-Rad). All reactions were performed in triplicate, and values within each triplicate that differed ≥0.5 CT were removed. The average of the remaining values was calculated for each sample using the Livak method (2-ΔΔCT) (17) with GADPH serving as the endogenous control. Changes in gene expression were represented as fold changes relative to the CON group.

### Microbiota and metabolite characterization

To profile the effects of GF2F diet on the gut microbial community structure, we assessed cecal and fecal microbial composition and metabolites from CON- and GF2F-fed mice as well as in feces from mice fed either 2′FL or scGOS. Acetic acid, propionic acid, and butyric acid were measured in feces and cecum content (CC) by gas chromatography as described previously (18). Data are expressed in mmol/g sample. Total genomic DNA was extracted from fecal samples (collected at day 30) and CC (collected at day 31) utilizing the FastDNA bead-beating Spin Kit for Soil (MP Biomedicals), according to the manufacturer's protocol. DNA concentrations were measured via fluorometric quantitation (Qubit; Life Technologies). Genomic DNA was prepared for microbiome amplicon sequencing using a 2-stage PCR protocol employing primers targeting the microbial small subunit ribosomal RNA (SSU or 16S rRNA) gene, as described previously [Naqib et al. 2018 (19)]. Briefly, primers CS1_515FB (ACACTGACGACATGGTTCTACAGTGYCAGCMGCCGCGGTAA) and CS2_806RB (TACGGTAGCAGAGACTTGGTCTGGACTACNVGGGTWTCTAAT) targeting the V4 variable region of the 16S rRNA gene were used for PCR (20). These amplicons were then prepared for high-throughput sequencing through a second 8-cycle PCR reaction with primers containing Illumina sequencing adapters, sample-specific barcodes, and “common sequences” (CS1 and CS2) at the 3′ ends. Negative control (no DNA) samples were included with each set of amplifications and did not indicate PCR contamination. Samples were pooled and purified according to the AMPure XP cleanup protocol (0.6×, vol/vol, Agencourt; Beckmann-Coulter) (21). The pooled libraries, with a 20% phiX spike-in, were loaded onto an Illumina MiniSeq mid-output flow cell (2 × 153 paired-end reads) and sequenced with the use of Fluidigm sequencing primers. Based on the distribution of reads per barcode, the amplicons (before purification) were repooled to generate a more balanced distribution of reads. The repooled and repurified libraries were then sequenced on a high-output MiniSeq run (2 × 153 paired-end reads). Library preparation, pooling, and sequencing were performed at the University of Illinois at Chicago Sequencing Core. Raw sequence data (FASTQ files) were deposited in the National Center for Biotechnology Information Sequence Read Archive, under the BioProject identifier PRJNA453755.

Raw FASTQ files for each sample were merged with the use of the software package PEAR version 0.9.8 (paired-end-read merger) (17, 22). Merged reads were quality trimmed, and primer sequences removed. Sequences <250 bases were discarded (CLC Genomics Workbench version 10.0; CLC Bio, Qiagen). Sequences were screened for chimeras (usearch8.1 algorithm) (23), and putative chimeric sequences were removed from the dataset (QIIME version 1.8) (24). Each sample was rarefied (22,000 sequences/sample) and data were pooled, renamed, and clustered into operational taxonomic units (OTUs) at 97% similarity (usearch8.1 algorithm). Representative sequences from each OTU were extracted and classified with the use of the uclust consensus taxonomy assigner (Greengenes 13_8 reference database). A biological observation matrix (BIOM) (25) was generated at each taxonomic level from phylum to species (“make OTU table” algorithm), and analyzed and visualized with the software packages Primer7 (26) and the R programming environment (27).

α-Diversity indices (within-sample) and β-diversity indices (between-sample) were used to examine changes in microbial community structure between mice group samples. α-Diversity indices (i.e., Shannon, richness, and evenness) were generated using the package “vegan” implemented in the R programming language. To examine β-diversity differences in microbial community composition between samples, the pairwise Bray-Curtis dissimilarity (nonphylogenetic) metric was generated with the Primer7 software package and used to perform analysis of similarity (ANOSIM) calculations. ANOSIM was performed at the taxonomic level of genus, using square-root-transformed data. The spatial patterns of microbial community structure by diet were summarized with the use of nonmetric multidimensional scaling (nMDS), and the routine bootstrap average on Bray-Curtis similarity matrices from microbial genus-level abundance data (28). The bootstrap average plots were overlain with 95% region estimates fitted to the bootstrap averages.

β-Diversity differences in relative abundance (RA) of individual taxa, between mice group samples, were assessed for significance using the Kruskal-Wallis test controlling for false-discovery rate, implemented within the software package QIIME (24). Taxa with an average abundance of <1% across the sample set were removed from the analysis. Microbial RA and Firmicutes/Bacteroidetes ratios between conditions were studied. The RA of individual taxa reported in our mouse model was accepted at a significance of *P* < 0.05 for false-discovery rate.

### Statistical analysis

All mouse variables were checked for normality in the software package SPSS version 22 (IBM) with the use of the Shapiro-Wilk normality test. Parametric one-way ANOVA, with Bonferroni's post-hoc test or nonparametric Mann-Whitney *U* test, and Kruskal-Wallis test, with Dunn's post-hoc test, were used to compare data between groups and are indicated in the figure legends. All microbial diversity data [e.g., richness, evenness, and Shannon index (29, 30) and RA of bacterial taxa (31, 32)] were exported, analyzed, and graphically presented as mean ± SEM.

Pearson correlations were applied to associate the different vaccine-specific response parameters (DTH, IgG1, IgG2a) with microbial metabolite metabolism and microbial community structure (RA of individual genera). These collective test results were considered statistically significant at *P* < 0.05. All data were analyzed with GraphPad Prism 7.0 software for Macintosh (GraphPad Software).

### Ethics statement

Experimental procedures were approved by the Ethics Committee of Animal Research of Utrecht University and the Central Commission for Animal Use (approvals DEC2015.II.243.038 and AVD108002016460) and complied with the principles of good laboratory animal care of the European directive for protection of animals used for scientific purposes.

## Results

### Enhanced cellular and humoral vaccination responses in GF2F mice

A significant increase in influenza-specific DTH response, measured as ear swelling and ear weight gain, was detected in vaccinated mice receiving the GF2F diet compared with those receiving the CON diet (Figure 1B: *P* < 0.01 for DTH; Figure 1C: *P* < 0.01 for ear weight increase). Vaccine-specific serum antibodies commonly used as a surrogate measure for vaccine efficacy were significantly higher in the serum of vaccinated mice receiving the GF2F diet, relative to those receiving the CON diet. Specifically, significantly higher levels of IgG1 (Figure 1D: *P* < 0.05) and IgG2a (Figure 1E: *P* < 0.05) were detected in the GF2F group relative to the CON diet group. These antibodies were not detected in nonvaccinated sham mice (data not shown).

### Local immune cell development in MLNs of GF2F mice

Antigen-presenting cells were characterized by staining of MHC-II, CD11c, and CD103 (Figure 2A). No differences were detected in the percentage of CD11c+ MHC-II+ cells (Figure 2B) or CD103+ DCs (Figure 2C) between dietary intervention groups. Next, the expression [median fluorescence intensity (MFI)] of costimulatory molecules CD86 and CD40 on migratory DCs in the MLNs were analyzed to determine the activation status of CD103+ DCs. A significant increase in the MFI of CD86 expression (*P* < 0.05) was observed between the CD103+ DCs of MLNs from GF2F mice compared with CON mice; no such effect was observed in the MFI of CD40 expression in CD103+ DCs (Figure 2D). The alteration of DCs in GF2F mice could influence B cell and antigen-specific T cell differentiation. To determine the impact of dietary intervention on local B cell responses, the percentage of activated B cells within the total B cell populations (CD19+ B220+ cells) was determined by CD27 staining (the gating strategy is shown in Figure 2E). No effect on the total B cell pool was detected by the dietary intervention (data not shown). A small (0.3%) but significantly (*P* < 0.05) higher percentage of CD27+ B cells was detected within MLNs of GF2F mice relative to CON mice (Figure 2F), consistent with increased vaccine-specific IgG1 and IgG2a levels in serum of GF2F mice. As T cells are involved in inducing profound vaccination responses, we also examined regulatory T cell (Treg) and T-helper 1 (Th1) cells in MLNs by staining for intracellular transcription factor Foxp3 and Tbet, respectively (Figure 2G). No difference in the percentage of CD4+ T cells was detected between groups (Figure 2H). Within CD4+ T cell populations, levels of CD25+ Foxp3+ Tregs were 1.64% higher (Figure 2I; *P* < 0.05) and levels of CD69+ Tbet+ Th1 cells were 2.2% higher in GF2F mice than in CON mice (Figure 2J; *P* < 0.05).

### Expression levels of intestinal mucosal barrier genes are altered in GF2F mice

**FIGURE 3:**
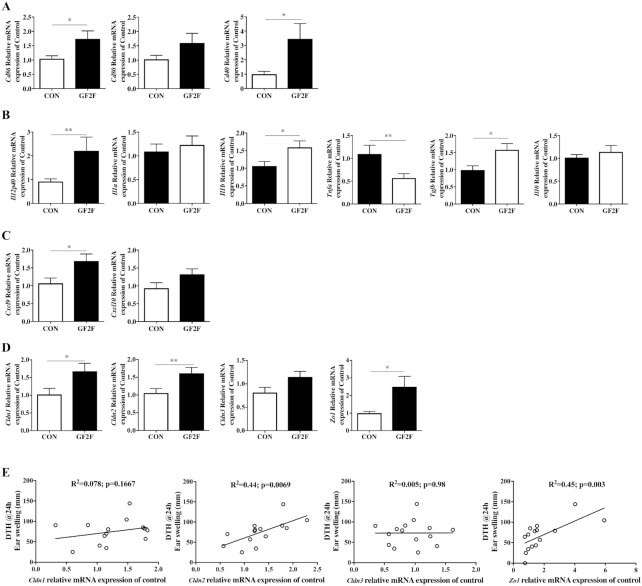
Effects of GF2F diet on the expression of (A) DC surface markers *Cd86, Cd80*, and *Cd40*; (B) cytokines *Il12p40, Il1a, Il1b, Tnfa, Tgfb*, and *Il10*; (C) chemokines *Cxcl9* and *Cxcl10*; and (D) tight junction–related genes *Cldn1, Cldn2, Cldn3*, and *Zo1* in the ileum of vaccinated mice. (E) Correlation between tight junction–related genes and DTH response. The Mann-Whitney *U* test was used for panels A–D. Data are presented as mean ± SEM for *n* = 7–9/group. Significantly different from CON: *P < 0.05; **P < 0.01. Pearson's correlation was used for panel (E). DC, dendritic cell; DTH, delayed type hypersensitivity; GF2F, scGOS/lcFOS/2′FL (2′FL, 2′-fucosyllactose; FOS, fructo-oligosaccharides; GOS, galacto-oligosaccharides; lc, long chain; sc, short chain); ns, not significant.

Locally induced immunologic changes within the intestinal mucosa were analyzed by qPCR on RNA extracted from ileum mucosal samples. These analyses revealed higher expression levels of *Cd86* (*P* < 0.05), *Cd80* (*P* = 0.11), and *Cd40* (*P* < 0.05) in GF2F mice relative to CON mice, suggesting alterations in the DC population (**Figure 3**A). Moreover, expression levels of several immunomodulatory cytokine and chemokine genes were significantly different between mice receiving the GF2F diet and CON diet. *Il12p40* (*P* < 0.01), *Il1b* (*P* < 0.05), *Cxcl9* (*P* < 0.05), and *Tgfb* (*P* < 0.05) were observed to be upregulated, whereas *Tnfa* (*P* < 0.01) was downregulated. No effect of diet was observed on expression levels of *Il1a, Il10*, and *Cxcl10* within the ileum (Figure 3B, C). The impact of dietary intervention on intestinal integrity was assessed through analysis of expression levels of tight junction genes including *Cldn1, Cldn2, Cldn3*, and *Zo1*. Expression levels of *Cldn1* (*P* < 0.05), *Cldn2* (*P* < 0.01), and *Zo1* (*P* < 0.05), but not *Cldn3*, were significantly higher in GF2F mice than in CON mice (Figure 3D). A correlation analysis across both groups revealed significant positive correlations between expression levels of *Cldn2* (*R*^2 ^= 0.44, *P* < 0.01) and *Zo1* (*R*^2 ^= 0.45, *P* < 0.01) with the DTH response (Figure 3E).

### SCFA concentrations are positively correlated with vaccine-specific DTH responses

**FIGURE 4:**
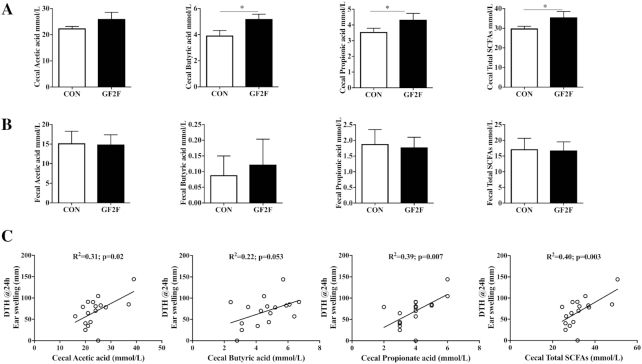
Effects of GF2F diet on (A) SCFA concentrations in cecal content (CC) and (B) feces of vaccinated mice, and (C) significant correlations with DTH responses. Feces were collected at day 30, and the cecum was collected at day 31 after killing the mice in order to measure the SCFAs. The absolute amounts of acetic acid, propionic acid, butyric acid, and SCFA mixture (acetic acid + propionic acid + butyric acid) are presented as mean ± SEM for *n* = 8–9/group. Statistical analysis with the Mann-Whitney *U* test was performed for panels A and B; *significantly different from CON, *P* < 0.05. Pearson's correlation analysis was performed for panel (C). CC, cecum content; DTH, delayed type hypersensitivity; GF2F, scGOS/lcFOS/2′FL (2′FL, 2′-fucosyllactose; FOS, fructo-oligosaccharides; GOS, galacto-oligosaccharides; lc, long chain; sc, short chain).

Total SCFA concentrations in the cecum were higher (*P* < 0.05) in GF2F mice than in CON mice. This was reflected by increases in butyric (*P* < 0.05) and propionic (*P* < 0.05) but not acetic acid levels (**Figure 4**A). In addition, SCFAs in fecal samples were assessed as a reflection of SCFA utilization and absorption. No differences in fecal SCFA concentrations were detected between the GF2F and CON diet groups (Figure 4B). Correlation analyses across both groups were conducted to identify possible relationships between cecal SCFA levels (microbial metabolism) and DTH, serum IgG1, or IgG2a (vaccine-specific immune response). DTH responses were correlated with cecal acetic acid (*R*^2^ = 0.31, *P* < 0.05), propionic acid (*R*^2^ = 0.39, *P* < 0.01), and total SCFA levels (*R*^2^ = 0.40, *P* < 0.01) (Figure 4C). Correlations between butyric acid and DTH trended towards significance (*R*^2^ = 0.22, *P* = 0.05) (Figure 4C), whereas significant correlations between butyric acid and vaccine-specific IgG1 (*R*^2^ = 0.39, *P* < 0.01) and IgG2a (*R*^2^ = 0.23, *P* < 0.05) were observed.

### SCFAs contribute to DC maturation and vaccine-specific T cell proliferation in vitro

**FIGURE 5:**
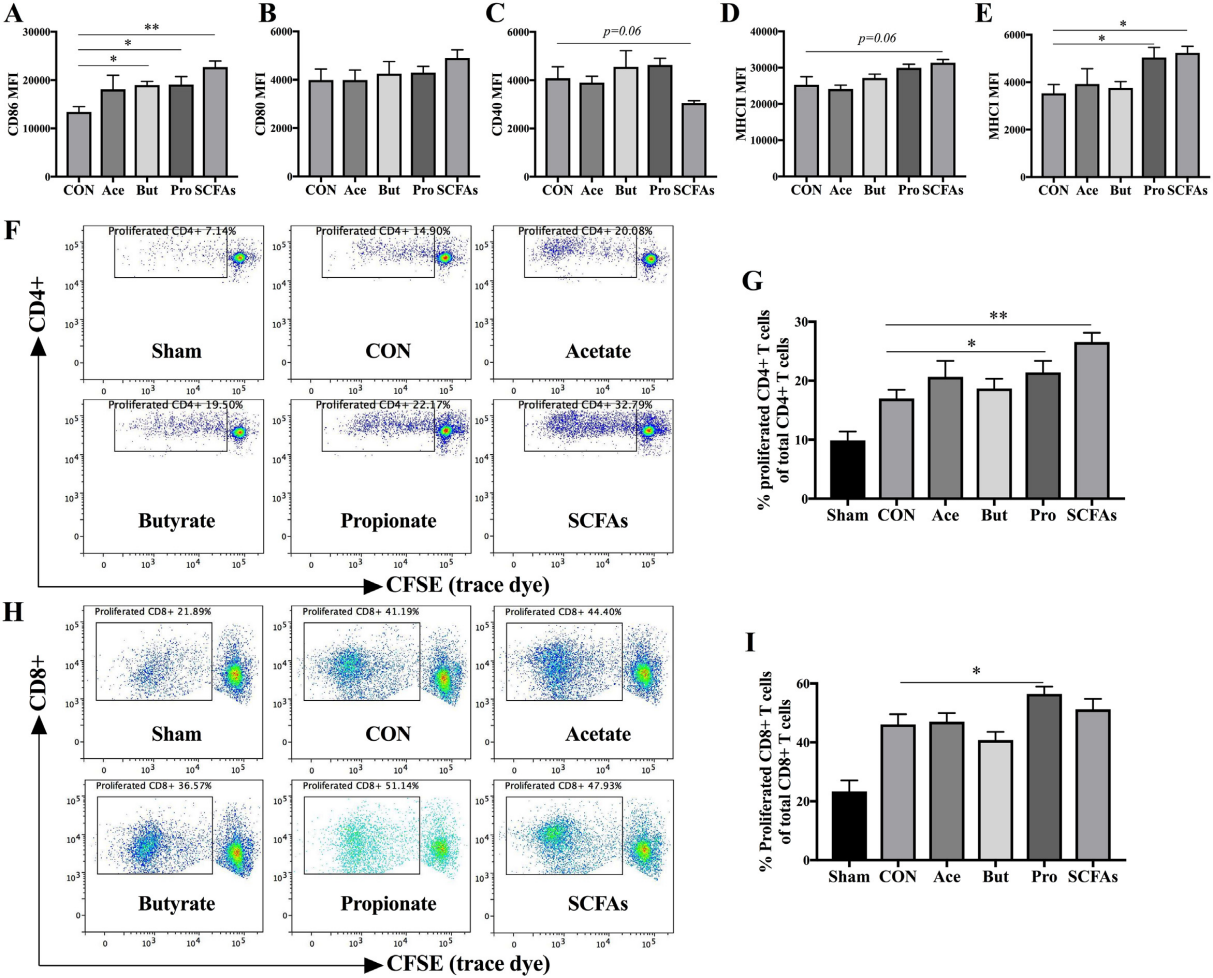
Effects of SCFAs on the expression of surface markers (A) CD86, (B) CD80, (C) CD40, (D) MHC-II, and (E) MHC-I, and percentage of proliferated (G) CD4+ and (I) CD8+ T cells after ex vivo restimulation. Maturation status of different treated BMDCs was distinguished based on their expression (MFI) of surface markers. Representative plots of proliferated (F) CD4+ and (H) CD8+ T cell after ex vivo restimulation by different BMDCs. The Kruskal-Wallis nonparametric test, followed by Dunn's post-hoc test for selected pairs, was used for panels A–E, G, and I. Data are presented as mean ± SEM, *n* = 4 for panels A–E; for panels G and I, spleens were obtained from 3 nonvaccinated (sham) or 6 vaccinated mice; BMDCs were obtained from 3 donor mice. Significantly different from CON: *P < 0.05; **P < 0.01. Ace, acetate; BMDC, bone marrow–derived dendritic cell; But, butyrate; CON, control; MFI, medium fluorescence intensity; Pro, propionate.

SCFAs have been shown to be potent modulators of antigen-presenting DCs (12), and we observed that the GF2F diet induced higher concentrations of specific SCFAs and that these compounds were significantly correlated with measures of immune responsiveness. To assess the direct effect of SCFAs on vaccine-specific immunity, we conducted an in vitro experiment where BMDCs were treated with acetate, butyrate, and propionate individually and as a mixture. An increase in the expression of the costimulatory molecule CD86 was detected in BMDCs treated with propionate, butyrate, and combined SCFAs (**Figure 5**A; *P* < 0.05, *P* < 0.05, and *P* < 0.01, respectively). No significant changes were detected in the expression of CD80 (Figure 5B) in any SCFA treatment, whereas trends towards decreased expression of CD40 were detected (Figure 5C; *P* = 0.06), and increased expression of MHC-II (Figure 5D; *P* = 0.06) was observed in BMDCs treated with the SCFA mixture. MHC-I expression on BMDCs was significantly elevated in BMDCs when pretreated with either propionate alone or with the combined SCFAs (Figure 5E; *P* < 0.05 and *P* < 0.05, respectively). An ex vivo restimulation assay was performed to determine the vaccine-specific antigen-presenting capacity of these BMDCs. Specifically, BMDCs were treated with SCFAs as described above, and subsequently loaded with or without 0.9 μg/mL vaccine (Influvacc) for 24 h before being cocultured with fresh whole splenocytes from nonvaccinated (sham) or influenza-vaccinated mice (receiving CON diet). The proliferation of vaccine-specific CD4+ and CD8+ T cells was determined by flow cytometry with CFSE labeling (Figure 5F, H). Treatments of BMDCs with propionate alone or with the SCFA mixture induced 10.5% and 16.6% higher vaccine-specific CD4+ T cell proliferation related to untreated BMDCs (Figure 5G; *P* < 0.05 and *P* < 0.01, respectively). No significant stimulation of CD4+ T cell proliferation was detected in treatments with acetate or butyrate alone. A 10.3% higher vaccine-specific CD8+ T cell proliferation was detected in BMDCs treated with propionate than in untreated BMDCs (Figure 5I; *P* < 0.05).

### Effect of GF2F diet on microbial community structure

**FIGURE 6:**
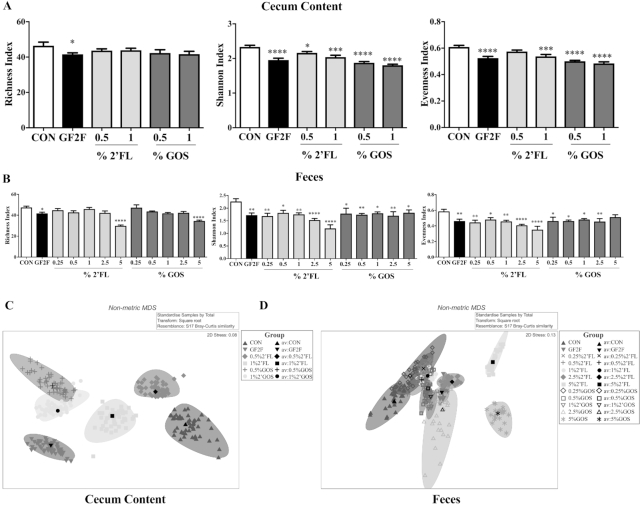
Impact of GF2F diet on α-diversity indices in the (A) cecal content (CC) and (B) feces, and β-diversity overall microbial community structure at the taxonomic level of genus in CC (C) and feces (D) of vaccinated mice. Diversity indices (Shannon, richness and evenness) are depicted at the taxonomic level of genus. Data are presented as mean ± SEM, *n* = 8–9/group. Significantly different from CON: *P < 0.05; **P < 0.01; ***P < 0.001; ^****^P < 0.0001. One-way analysis of variance test for parametric data and Bonferroni's post-hoc test were used in panel A. The Kruskal-Wallis test for nonparametric data and Dunn's post-hoc test were used in panel B. Nonmetric multidimensional scaling of bootstrap averages for the CON and treatment groups in CC (C) and feces (D). Colored ovals represent the 95% region estimates for the mean communities in each treatment group. Black symbols represent the group means of the repeated bootstrap averages. ANOSIM is used to assess statistical significance of divergent microbial community structure (Supplemental Table 2). ANOSIM, analysis of similarity; CC, cecum content; CON, control; GF2F, scGOS/lcFOS/2′FL (2′FL, 2′-fucosyllactose; FOS, fructo-oligosaccharides; GOS, galacto-oligosaccharides; lc, long chain; sc, short chain).

α-Diversity indices were calculated as: Shannon index (H′ = –∑ sum[Pi/log(Pi)], where Pi = RA of each taxon), Pielou's evenness [J′ = H′/log(S), where S = number of taxa present in each sample], and richness (number of taxa present in each sample). Microbial richness in cecal (**Figure 6**A) and fecal samples (Figure 6B) was significantly lower in GF2F mice than in CON mice (*P* ˂ 0.05). Additionally, microbial richness in fecal samples from mice receiving 2′FL (5%) or scGOS (5%) was significantly lower (*P* ˂ 0.0001) than that of CON mice (Figure 6). Additionally, microbial richness in fecal samples from mice receiving 2′FL (5%) or scGOS (5%) was significantly lower ( P ˂ 0.0001) than that of CON mice (Figure 6B). Similarly, Shannon index values were significantly lower in cecal and fecal samples from all dietary intervention groups than in those from CON mice. Decreased evenness in microbial communities was also observed in fecal and cecum samples from all dietary intervention groups relative to CON (Figure 6A, B, respectively). A strong effect of dietary intervention was observed in cecal and fecal microbiome analysis (Figure 6C, D, respectively) with significant differences in microbial community structure between cecal and fecal samples across all dietary groups, and between dietary groups in cecal and fecal samples (except for the 0.25% GOS dietary intervention in feces), as assessed by ANOSIM (**Supplemental Table 2**). Cecal and fecal samples from GF2F mice had the greatest divergence from CON mice relative to the other dietary interventions (cecal ANOSIM global *R* = 0.872, *P* < 0.0001; fecal: global *R* = 0.600, *P* < 0.0001).

**FIGURE 7:**
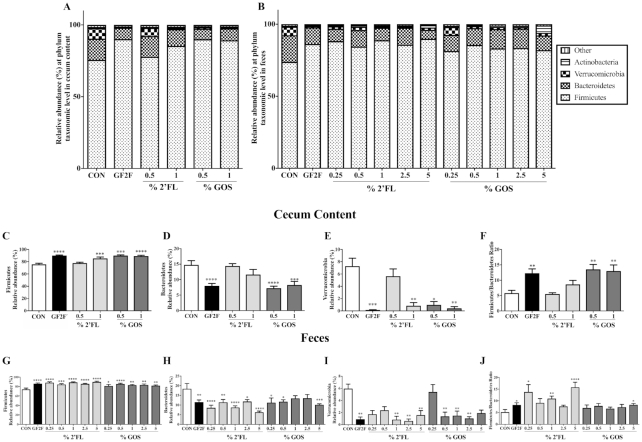
Impact of GF2F diet in both (A) cecal (CC) and (B) fecal microbial compositions, and RA (%) of 3 major bacterial phyla in the (C–E) CC and (G–I) feces at the taxonomic level of phylum. Firmicutes/Bacteroidetes ratios in the (F) CC and (J) feces from CON and different dietary intervention groups. The Kruskal-Wallis test for nonparametric data and Dunn's post-hoc test were used for comparisons in panels C–J. Data are presented as mean ± SEM, *n* = 8–9/group. Significantly different from CON: *P < 0.05; **P < 0.01; ***P < 0.001; ^****^P < 0.0001. CC, cecum content; CON, control; GF2F, scGOS/lcFOS/2′FL (2′FL, 2′-fucosyllactose; FOS, fructo-oligosaccharides; GOS, galacto-oligosaccharides; lc, long chain; sc, short chain); RA, relative abundance.

**FIGURE 8:**
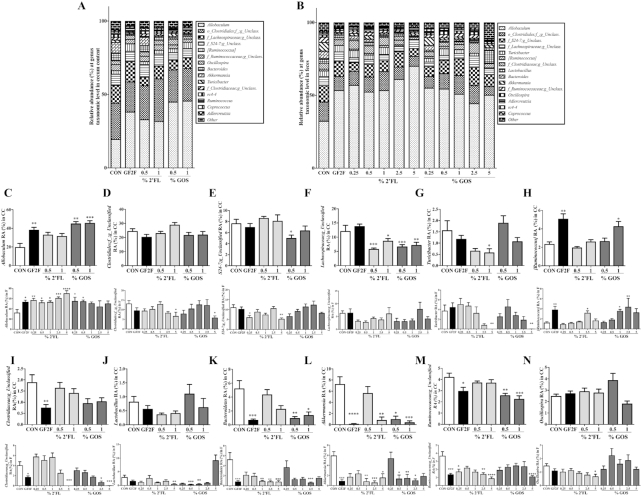
Impact of GF2F diet in both (A) cecal (CC) and (B) fecal microbial compositions, and RA (%) of 12 individual bacterial genera in the (C–N) CC and feces at the genus taxonomic level. The Kruskal-Wallis test for nonparametric data and Dunn's post-hoc test were used for comparisons in panels C–N. Data are presented as mean ± SEM, *n* = 8–9/group. Significantly different from CON: *P < 0.05; **P < 0.01; ***P < 0.001; ^****^P < 0.0001. CC, cecum content; CON, control; GF2F, scGOS/lcFOS/2′FL (2′FL, 2′-fucosyllactose; FOS, fructo-oligosaccharides; GOS, galacto-oligosaccharides; lc, long chain; sc, short chain) RA, relative abundance.

Dietary intervention led to significantly altered microbial community structures in cecal and fecal samples, and this difference was manifested at multiple taxonomic levels, including phylum (**Figure 7**A, B) and genus (**Figure 8**A, B). At the phylum level, Firmicutes, Bacteroidetes, and Verrucomicrobia were the most abundant taxa in both cecal and fecal samples across all dietary treatments (Figure 7A, B). The RA of bacteria from the phylum Firmicutes was significantly increased in cecal samples from GF2F (*P* < 0.0001), 1% 2′FL (*P* < 0.001), 0.5% scGOS (*P* < 0.001), and 1% scGOS (*P* < 0.0001) treatments relative to CON (Figure 7C). The increased RA of Firmicutes in the cecum was associated with the increased RAs of bacterial genera *Allobaculum* (Figure 8C; *P* < 0.01 for GF2F, *P* < 0.01 for 0.5% scGOS, and *P* < 0.001 for 1% scGOS), unclassified *Lachnospiraceae* (Figure 8F; *P* < 0.01 for GF2F, *P* < 0.001 for 0.5% scGOS, and *P* < 0.001 for 1% scGOS), and *Lachnospiraceae* and *Ruminococcus* (Figure 8H; *P* < 0.01 for GF2F, and *P* < 0.05 for 1% scGOS). However, the cecal samples showed a decreased RA of genera unclassified *Clostridiaceae* (Figure 8I; *P* < 0.01 for GF2F) and *Ruminococcaceae* (Figure 8M; *P* < 0.05 for GF2F, *P* < 0.01 for 0.5% scGOS, and *P* < 0.001 for 1% scGOS). At the genus level, the increased RA of Firmicutes was attributed to bacterial genera *Allobaculum* (Figure 8C; *P* < 0.05 for GF2F, *P* < 0.01 for 0.25% 2′FL, *P* < 0.05 for 0.5% 2′FL, *P* < 0.05 for 1% 2′FL, *P* < 0.001 for 2.5% 2′FL, *P* < 0.0001 for 5% 2′FL, *P* < 0.05 for 0.25% scGOS, and *P* < 0.05 for 1% scGOS) and *Lachnospiracea*and *Ruminococcus* (Figure 8H; *P* < 0.01 for GF2F, *P* < 0.05 for 2.5% 2′FL, *P* < 0.05 for 1% scGOS, and *P* < 0.01 for 2.5% scGOS). A decrease in unclassified *Clostridiaceae* (Figure 8I; *P* < 0.01 for GF2F, *P* < 0.001 for 5% 2′FL, *P* < 0.05 for 2.5% scGOS, and *P* < 0.001 for 5% scGOS), *Lactobacillus* (Figure 8J; *P* < 0.01 for 5% 2′FL, *P* < 0.001 for 0.5% scGOS, *P* < 0.01 for 1% scGOS), unclassified *Ruminococcaceae* (Figure 8M; *P* < 0.001 for GF2F, *P* < 0.05 for 2.5% 2′FL, *P* < 0.0001 for 5% 2′FL, and *P* < 0.0001 for 5% scGOS), and *Oscillospira* (Figure 8N; *P* < 0.05 for 5% 2′FL) was observed in the fecal samples relative to CON mice.

The second most abundant phylum was Bacteroidetes, which was significantly decreased in GF2F (*P* < 0.0001) and scGOS (*P* < 0.0001 and *P* < 0.001 for 0.5% and 1% scGOS, respectively) relative to CON (Figure 7D). Changes in RA were primarily driven by Bacteroidetes (Figure 8K: *P* < 0.001 for GF2F, *P* < 0.01 and *P* < 0.05 for 0.5% and 1% scGOS, respectively) and unclassified *S24–7* (Figure 8E; *P* < 0.05 for 0.5% scGOS). No significant effects of 2′FL on the RA of Bacteroidetes and corresponding genera in the cecal samples were observed (Figure 8E). Conversely, a reduction of Bacteroidetes was observed in GF2F (*P* < 0.01), scGOS (*P* < 0.05, *P* < 0.05, and *P* < 0.001 for 0.25%, 0.5%, and 5% scGOS, respectively) and 2′FL (*P* < 0.0001, *P* < 0.01, *P* < 0.0001, *P* < 0.05, and *P* < 0.001 for 0.25%, 0.5%, 1%, 2.5%, and 5% 2′FL, respectively) in fecal samples relative to CON (Figure 7H). Decreases were attributable to Bacteroidetes (*P* < 0.001, *P* < 0.01, *P* < 0.01, *P* < 0.001, and *P* < 0.001 for GF2F, 1%, 2.5%, and 5% 2′FL, and 2.5% scGOS, respectively) (Figure 8K).

The third most abundant phylum was Verrucomicrobia, which was significantly lower in cecal samples of GF2F (*P* < 0.001), 1% 2′FL (*P* < 0.01), 0.5% scGOS (*P* < 0.05), and 1% scGOS (*P* < 0.01) relative to CON (Figure 7E), which was associated with *Akkermansia* (Figure 8L; *P* < 0.0001 for GF2F, *P* < 0.01 for 1% 2′FL, *P* < 0.05 for 0.5% scGOS, and *P* < 0.001 for 1% scGOS). In the fecal samples a lower abundance of Verrucomicrobia was detected in GF2F (*P* < 0.01), 1% 2′FL (*P* < 0.01), 2.5% 2′FL (*P* < 0.01), 5% 2′FL (*P* < 0.01), 0.5% scGOS (*P* < 0.01), 1% scGOS (*P* < 0.01), and 2.5% scGOS (*P* < 0.01) relative to CON (Figure 7I), which was attributed to *Akkermansia* (Figure 8L; *P* < 0.01 for GF2F, *P* < 0.01 for 1% 2′FL, *P* < 0.001 for 2.5% 2′FL, *P* < 0.05 for 5% 2′FL, *P* < 0.05 for 0.5% scGOS, *P* < 0.01 for 1% scGOS, and *P* < 0.05 for 2.5% scGOS). In addition, a significant increase in the Firmicutes/Bacteroidetes ratio, which changes with age (33) was observed in cecal samples of GF2F (*P* < 0.01), scGOS (*P* < 0.01 and *P* < 0.01 for 0.5% and 1% scGOS, respectively) relative to CON mice (Figure 7F) as well as fecal samples in GF2F (*P* < 0.05), 0.25% 2′FL (*P* < 0.05), 1% 2′FL (*P* < 0.01), 5% 2′FL (*P* < 0.0001), and 5% scGOS (*P* < 0.05) relative to CON mice (Figure 7J).

### Correlation between microbial taxa and enhanced vaccine-specific responses

**FIGURE 9:**
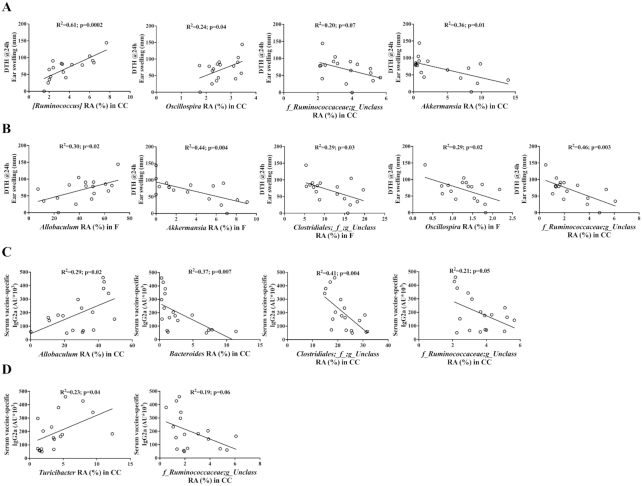
Significant Pearson's correlations between specific genera in the (A) cecum (CC) or (B) feces and DTH, and significant correlations between specific genus in the (C) CC or (D) feces and serum IgG2a level. No significant correlations were found between specific genera and serum IgG1. Pearson's correlation analysis was conducted to analyze correlations between DTH or IgG2a and significantly changed genus in the CC and feces. CC, cecum content; DTH, delayed type hypersensitivity.

Following the hypothesis that changes in gastrointestinal microbiome composition can affect mucosal responsiveness and thereby influence vaccination responsiveness, we searched for correlations between immune markers and individual microbial taxa across the GF2F and CON mice. Correlation analysis revealed positive correlations between DTH and RA of the bacterial genera *Ruminococcus* and *Oscillospira* in cecal samples (**Figure 9**A; *R*^2 ^= 0.61, *P* < 0.001; *R*^2 ^= 0.24, *P* < 0.05, respectively), and the genus *Allobaculum* in feces (Figure 9B: *R*^2 ^= 0.30, *P* < 0.05). Negative correlations were found between the RA of the bacterial genera *Akkermansia* in cecal samples (Figure 9A; *R*^2 ^= 0.36, *P* < 0.05), and the genera *Akkermansia, Oscillospira*, and unclassified *Clostridiales* and *Ruminococcaceae* in feces (Figure 9B; *R*^2 ^= 0.44, *P* < 0.01; *R*^2 ^= 0.29, *P* < 0.05; *R*^2 ^= 0.29, *P* < 0.05; *R*^2 ^= 0.36, *P* < 0.01, respectively). Positive correlations were observed between serum IgG2a and the RA of the genus *Allobaculum* in in cecal samples (Figure 9C; *R*^2 ^= 0.29, *P* < 0.05) and *Turicibacter* in feces (Figure 9D; *R*^2 ^= 0.23, *P* < 0.05). Significant negative correlations were measured between serum IgG2a and the taxa *Bacteroides* and unclassified *Clostridiales* and *Ruminococcaceae* in cecal samples (Figure 9C; *R*^2 ^= 0.37, *P* < 0.01; *R*^2 ^= 0.41, *P* < 0.01; *R*^2 ^= 0.21, *P* = 0.05, respectively), and unclassified *Ruminococcaceae* in feces (Figure 9D; *R*^2 ^= 0.19, *P* = 0.06).

## Discussion

During the first years of life, an infant's immune system remains under development, as it is still learning to deal with complex host-microbe interactions at mucosal surfaces. Specific prebiotic oligosaccharides, such as scGOS/lcFOS, influence microbiome development, but more importantly reduce the development of allergies and limit the impact of pediatric infections (34, 35). It has been shown that diets containing 2′FL lead to improved cellular and humoral immune responses (14). In the present study, we demonstrate that a dietary intervention with a combination of 2′FL and scGOS/lcFOS, more effectively enhances mucosal immune responsiveness to influenza vaccination. Specifically, we demonstrate that a GF2F diet–induced improvement in vaccine responses is modulated via intestinal mucosal sites by inducing profound changes within immune cells, microbial composition, and metabolites. This reveals a key role for microbiota in promoting immunity to vaccinations. Our findings may provide for a novel and effective strategy to improve the vaccine responsiveness by targeting the mucosal immune system.

Nutritional interventions, leading to improved Th1 responsiveness, contribute to protective immunity against viral infections, the highest contraction rates of which occur in early infancy (36, 37). In addition to our previous studies showing that specific prebiotics enhance systemic immunity (14, 15), we now demonstrate that the GF2F diet can stimulate local B cell activation and Th1 cells within the MLN (14), supporting immune development through the gut (Figure 2). The increase in the percentage of activated B cells, although small, coincides with the higher levels of vaccine-specific plasma IgG1 and IgG2a detected in mice receiving the GF2F diet compared with the CON mice. The increased frequency of Tbet+ Th1 cells, and elevated expression levels of *Il12p40* and *Il1b* observed in the GF2F mice, implies an upregulation in Th1 responsiveness, which can be related to the increased vaccine-specific DTH response. Interestingly, the increased percentage of Tregs within the MLN underpins the regulation of gut homeostasis, which was amplified by the effect of the tight junction genes *Cldn1, Cldn2*, and *Zo1*, although the actual intestinal barrier function has not been studied. The positive correlation between DTH and *Cldn2*, a small molecule transport regulator which may facilitate DCs in penetrating gut epithelial monolayers and sampling antigens more efficiently, suggests a significant role between the intestinal barrier and immune response development. However, a comprehensive study on the individual role of tight-junction regulators combined with DC development and function is necessary to fully understand these interactions.

It is known that 2′FL, as well as scGOS/lcFOS, can directly modulate DCs and subsequent T cell responses in vitro (14, 38). Our findings further support the role of direct immunomodulation by GF2F contributing to observed enhanced vaccine responses in vivo. Indirect effects of the dietary interventions arise by way of modulation of the gut microbiota composition and metabolic function. Commensal bacteria can influence vaccine-specific immune responses (5, 8, 39, 40), and the metabolites they produce (SCFAs) can support optimal antibody responses (11, 41). Indeed, the dietary interventions induced a significant overall change in microbial community structure in both fecal and cecal samples relative to samples from control mice. Although a high α-diversity of the gut microbiota can be beneficial in certain disease contexts such as autoimmune diabetes (42) and allergic disorders (43, 44), the GF2F diet seems to influence the vaccine response by selectively stimulating growth of specific bacteria, which in turn has a negative impact on α-diversity parameters of the gut microbiota. A lower α-diversity of breastfed infants was consistent with the enrichment of genes required for the degradation of HMOS, parallel to changes observed with the GF2F diet compared with the CON diet. Nonetheless, increased α-diversity may offer a benefit in promoting the health of the host later in life when the diet is more diverse (45).

Microbial metabolites such as SCFAs possess immunomodulatory properties that work on various cell types including DCs, B cells, and T cells (38, 46, 47). The increased levels of SCFAs detected in the cecal content of mice fed the GF2F diet suggest possible modulation of B cells and DCs in our model. Consistent with previous findings that SCFAs regulated B cell functionality to boost optimal antibody responses (11, 41), elevated antibody levels were observed in mice fed GF2F. Furthermore, butyric acid concentrations detected within the cecal samples were positively correlated with serum IgG1 and IgG2a levels, suggesting that GF2F can enhance the humoral responses via microbial synthesis of SCFAs. Interestingly, within in vitro systems we further identified that increased propionic acid supported ex vivo proliferation of influenza-specific CD4+ and CD8+ T cells (Figure 5). These findings support the hypothesis that GF2F, via bacterial metabolites, can modulate both phenotype and function of antigen-presenting DCs, consequently improving immune development and enhancing the vaccine response. This indicates the importance of providing diversity within oligosaccharide structures to optimally induce immune development on both B and T cell compartments until full protection is acquired. The vaccination model used in the current study is well suited to establish the capacity of the immune system to respond to a given antigen (48) and allowed us to study immunomodulation by nutritional intervention with GF2F, providing a robust understanding of nutritional interventions that can be translated easily for clinical trials.

In summary, this study addressed the link between changing gut microbial community structure and metabolites, thereby improving vaccine-specific immune responses. This study provides a novel strategy for the optimization of vaccine efficacy. In addition, the observed beneficial effects of GF2F may improve the immune system by optimizing gut microbiota composition and metabolic function.

## Supplementary Material

nxz006_Supplemental_File

## Notes

Supported by China Scholarship Council (CSC) award 201406050044 to LX.

Author disclosures: JG is head of the Division of Pharmacology, Utrecht Institute for Pharmaceutical Sciences, Faculty of Science at the Utrecht University, and partly employed by Nutricia Research. Both BS and BL are employed by Nutricia Research. BL, as indicated by the affiliations, is leading a strategic alliance between University Medical Centre Utrecht/Wilhelmina Children's Hospital and Nutricia Research. LX, PA, TL, IA, BS, SO, JG, AN, SG, AK, GF, and BL report no conflicts of interest.

Supplemental Tables 1 and 2 are available from the “Supplementary data” link in the online posting of the article and from the same link in the online table of contents at https://academic.oup.com/jn/.

Abbreviations used: ANOSIM, analysis of similarity; BMDC, bone marrow–derived dendritic cell; CC, cecum content; CON, control; DC, dendritic cell; DTH, delayed type hypersensitivity; FOS, fructo-oligosaccharides; Foxp3, forkhead box protein 3; GF2F, scGOS/lcFOS/2′FL; GOS, galacto-oligosaccharides; HMOS, human milk oligosaccharides; iDC, immature BMDC; lc, long chain; MFI, median fluorescence intensity; MLN, mesenteric lymph node; OTU, operational taxonomic unit; RA, relative abundance; sc, short chain; Th1, T-helper 1; Treg, regulatory T cell; 2′FL, 2′-fucosyllactose.
