# Supplementary material for: The Combination of 2′-Fucosyllactose with Short-Chain Galacto-Oligosaccharides and Long-Chain Fructo-Oligosaccharides that Enhance Influenza Vaccine Responses Is Associated with Mucosal Immune Regulation in Mice
Source: J Nutr. 2019 Apr 24;149(5):856–69. doi: 10.1093/jn/nxz006 (PMC6499104; doi:10.1093/jn/nxz006)
Supplement: nxz006_Supplemental_File [file nxz006_supplemental_file.pdf]

Supplementary Data

Supplemental Table 1. Sequences of specific primers for detected genes with corresponding accession number.

Gene ID	Accession number	Forward primer sequence (5'-3')	Reverse primer sequence (5'-3')
Il12b	NM_001303244.1	GTAACCAGAAAGGTGCGTTCC	GAACACATGCCCACTTGCTG
Il1a	NM_010554.4	ATGAAGCTCGTCAGGCAGAAG	GAGATAGTGTTTGTCCACATCCTGAT
Il1b	NM_008084.2	ATCCCAAGCAATACCCAAAGAA	GCTGATGTACCAGTTGGGGAA
Cxcl9	NM_008599	TCTGCCATGAAGTCCGCTG	CAGGAGCATCGTGCATTCCT
Cxcl10	NM_021274.2	GCCGTCATTTTCTGCCTCAT	GCTTCCCTATGGCCCTCATT
Cd80	NM_009855.2	TGGGAAAAACCCCCAGAAGAC	GCCCGAAGGTAAGGCTGTT
Cd86	NM_019388.3	CAGCACGGACTTGAACAACC	CTCCACGGAAACAGCATCTGA
Cd40	NM_011611.2	ACTAATGTCATCTGTGGTTTAAAGT	GAAACACCCCGAAAATGGT
Cldn1	NM_016674	TCTACGAGGGACTGTGGATG	TCAGATTCAGCTAGGAGTCG
Cldn2	NM_016675.4	GGCTGTTAGGCTCATCCAT	TGGCACCAACATAGGAACTC
Cldn3	NM_009902	AAGCCGAATGGACAAAGAA	CTGGCAAGTAGCTGCAGTG
Zo1	NM_009386	CGAGGCATCATCCCAAATAAGAAC	TCCAGAAGTCTGCCCGATCAC
Tnfa	NM_013693.3	AACGGCATGGATCTCAAAGA	TTTCTCCTGGTATGAGATAGCAAATC
Tgfb	NM_009368.3	GCTGTTGAGGAGAGAGTCCA	TCCATTGGGCTGAAAGGTGT
GAPDH	NM_008084.2	GAACATCATCCCTGCATCC	CACATTGGGGGTAGGAACAC

Supplementary Data

Supplemental Table 2. Across-group analysis of similarity (ANOSIM) results for mice cecum content and feces microbiota compositions. Global *R* comparison was based on ANOSIM performed within the software package Primer7. *P*-values were calculated based on a permutational analysis, employing 999 permutations; square-root transformation analysis. CON = control; GF2F=scGOS/lcFOS/2'FL.

**Cecum content (CC) (Genus Taxonomic Level)**			**Feces (F) (Genus Taxonomic Level)**		
**Comparisons**	**Global *R***	***P*-value[a]**	**Comparisons**	**Global *R***	***P*-value[a]**
CON vs. GF2F	0.872	**<0.0001**	CON vs. GF2F	0.600	**<0.0001**
CON vs. 1% 2'FL	0.498	**<0.0001**	CON vs. 5% 2'FL	0.893	**<0.0001**
CON vs. 0.5% 2'FL	0.240	**<0.01**	CON vs. 2.5% 2'FL	0.766	**<0.0001**
CON vs. 1% GOS	0.814	**<0.0001**	CON vs. 1% 2'FL	0.634	**<0.0001**
CON vs. 0.5% GOS	0.775	**<0.0001**	CON vs. 0.5% 2'FL	0.250	**<0.01**
GF2F vs. 1% 2'FL	0.628	**<0.0001**	CON vs. 0.25% 2'FL	0.385	**<0.001**
GF2F vs. 0.5% 2'FL	0.965	**<0.0001**	CON vs. 5% GOS	0.906	**<0.0001**
GF2F vs. 1% GOS	0.360	**<0.0001**	CON vs. 2.5% GOS	0.435	**<0.001**
GF2F vs. 0.5% GOS	0.534	**<0.0001**	CON vs. 1% GOS	0.584	**<0.0001**
			CON vs. 0.5% GOS	0.53	**<0.001**
			CON vs. 0.25% GOS	0.182	**ns**
			GF2F vs. 5% 2'FL	0.728	**<0.0001**
			GF2F vs. 2.5% 2'FL	0.308	**<0.01**
			GF2F vs. 1% 2'FL	0.433	**<0.0001**
			GF2F vs. 0.5% 2'FL	0.420	**<0.0001**
			GF2F vs. 0.25% 2'FL	0.461	**<0.0001**
			GF2F vs. 5% GOS	0.866	**<0.0001**
			GF2F vs. 2.5% GOS	0.159	**<0.05**
			GF2F vs. 1% GOS	0.254	**<0.01**
			GF2F vs. 0.5% GOS	0.222	**<0.05**
			GF2F vs. 0.25% GOS	0.534	**<0.0001**
